# Ribosomal proteins and human diseases: molecular mechanisms and targeted therapy

**DOI:** 10.1038/s41392-021-00728-8

**Published:** 2021-08-30

**Authors:** Jian Kang, Natalie Brajanovski, Keefe T. Chan, Jiachen Xuan, Richard B. Pearson, Elaine Sanij

**Affiliations:** 1 Division of Cancer Research, Peter MacCallum Cancer Centre, Melbourne, VIC Australia; 2 Sir Peter MacCallum Department of Oncology, University of Melbourne, Melbourne, VIC Australia; 3 Department of Biochemistry and Molecular Biology, Monash University, Clayton, VIC Australia; 4 Department of Biochemistry and Molecular Biology, University of Melbourne, Melbourne, VIC Australia; 5 Department of Clinical Pathology, University of Melbourne, Melbourne, VIC Australia; 6 St. Vincent’s Institute of Medical Research, Fitzroy, VIC Australia

**Keywords:** Oncogenes, Cell biology

## Abstract

Ribosome biogenesis and protein synthesis are fundamental rate-limiting steps for cell growth and proliferation. The ribosomal proteins (RPs), comprising the structural parts of the ribosome, are essential for ribosome assembly and function. In addition to their canonical ribosomal functions, multiple RPs have extra-ribosomal functions including activation of p53-dependent or p53-independent pathways in response to stress, resulting in cell cycle arrest and apoptosis. Defects in ribosome biogenesis, translation, and the functions of individual RPs, including mutations in RPs have been linked to a diverse range of human congenital disorders termed ribosomopathies. Ribosomopathies are characterized by tissue-specific phenotypic abnormalities and higher cancer risk later in life. Recent discoveries of somatic mutations in RPs in multiple tumor types reinforce the connections between ribosomal defects and cancer. In this article, we review the most recent advances in understanding the molecular consequences of RP mutations and ribosomal defects in ribosomopathies and cancer. We particularly discuss the molecular basis of the transition from hypo- to hyper-proliferation in ribosomopathies with elevated cancer risk, a paradox termed “Dameshek’s riddle.” Furthermore, we review the current treatments for ribosomopathies and prospective therapies targeting ribosomal defects. We also highlight recent advances in ribosome stress-based cancer therapeutics. Importantly, insights into the mechanisms of resistance to therapies targeting ribosome biogenesis bring new perspectives into the molecular basis of cancer susceptibility in ribosomopathies and new clinical implications for cancer therapy.

## Introduction

Cell growth and proliferation are two distinct but coupled biological processes that are directly dependent on the tight coordination of protein synthesis and metabolic activity. These biological capabilities are essential characteristics that enable cancer cells to sustain uncontrolled proliferation.^1^ The biosynthesis of ribosomes, the molecular machines that translate messenger RNA (mRNA) into proteins,^2^ is a fundamental biological process that is intimately linked to cell growth and proliferation and is considered to be one of the most energy-consuming processes in proliferating mammalian cells.^3–6^

**Fig. 1:**
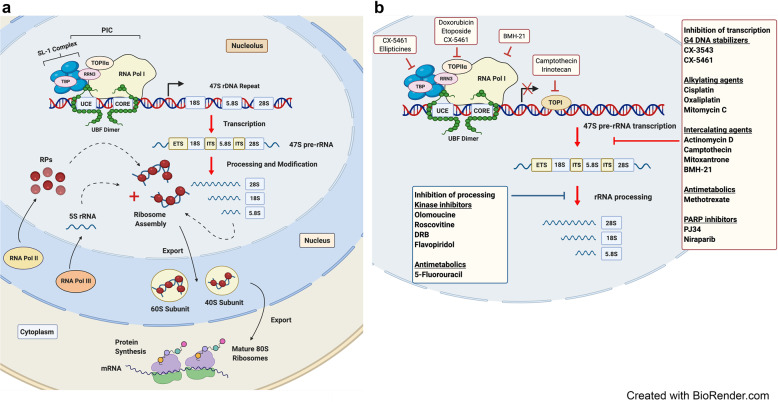
A schematic representation of ribosome biogenesis in mammalian cells. **a** Ribosome biogenesis is a tightly coordinated process involving all three RNA polymerases (Pol I, Pol II, and Pol III). RNA Pol I transcribes the ribosomal RNA (rRNA) genes (rDNA) to produce the 47S precursor rRNA (47S pre-rRNA) transcript in the nucleolus. Pol I transcription initiation involves binding of the upstream binding factor (UBF) to the core promoter region (core) and upstream control element (UCE) of the rDNA promoters and facilitating the recruitment and binding of the selectivity factor 1 (SL-1) complex. SL-1 is composed of the TATA-box-binding protein (TBP) and five Pol I-specific TATA-box-associated factors (TAFs). This complex in turn recruits the Pol I-specific initiation factor RRN3, which associates with DNA topoisomerase II*α* (TOPII*α*) and Pol I to complete assembly of a transcriptionally-competent Pol I complex. Following transcription, the 47S pre-rRNA is subsequently cleaved and processed into the mature 18S, 5.8S, and 28S rRNA species. These molecules are then assembled along with ribosomal proteins and the 5S rRNA produced by Pol II and III, respectively, to form the major catalytic and architectural components of the small (40S) and the large (60S) ribosomal subunits. Once assembled, ribosomal complexes are exported from the nucleolus to the cytoplasm, where they form the mature (80S) ribosome required to initiate mRNA translation and thus protein synthesis. **b** A diverse range of anticancer drugs target ribosome biogenesis by inhibiting Pol I transcription and/or pre-rRNA processing

Ribosome biogenesis is a highly dynamic and coordinated process, in which ribosomal RNA (rRNA) is synthesized, modified and assembled with RPs to form mature ribosomes. Ribosome biogenesis takes place within specialized subnuclear compartments known as the nucleoli^7,8^ and depends on the coordinated regulation of the three DNA-dependent RNA polymerases (Pol I, Pol II, and Pol III), as well as the involvement of a plethora of transcription factors, small nucleolar RNAs (snoRNAs) and non-RPs that cooperatively promote the transcription, modification and processing of rRNAs, synthesis of RPs and ribosome assembly^2,9–12^ (Fig. 1a). This process is exquisitely regulated by multiple signaling pathways in response to growth factors, energy and nutrients that together modulate protein synthesis and thereby cell growth rate and proliferation.^10,13–16^

Defects in ribosome biogenesis and function account for the pathogenesis of a heterogeneous group of diseases called ribosomopathies. Ribosomopathies are generally defined as diseases caused by mutations in RPs or factors associated with Pol I transcription and rRNA processing, resulting in the disruption of ribosome production or assembly.^17–21^ There is a growing list of diseases that have been classified as ribosomopathies; however, the extent to which defects in ribosome synthesis contribute to clinical phenotypes remains to be defined. In Treacher Collins Syndrome, Diamond–Blackfan anemia and Shwachman-Diamond syndrome, the defects in ribosome synthesis have been causally linked to disease pathotypes. However, in other putative ribosomopathies such as X-linked-dyskeratosis congenita and cartilage–hair hypoplasia–anauxetic dysplasia, Blooms and Werner syndrome and cohesinopathies, it is likely that stress responses associated with ribosomal defects and/or altered mRNA translation contribute to a component of their disease phenotype and impact on disease severity.^20^ Ribosomopathies are characterized by tissue-specific phenotypic abnormalities. For example, the hematopoietic system is frequently affected, which is thought to be due to tissue-specific sensitivity to p53 pathway activation in response to ribosome stress.^22^ Ribosomopathy patients also have a higher risk of developing cancer.^23^ The paradoxical transition from early symptoms due to hypo-proliferative phenotypes to an increased cancer risk later in life, was first reported by Dameshek in 1967 and referred to as Dameshek’s riddle.^24^ The mechanisms by which genetically compromised ribosome biogenesis leads to hyper-proliferative cancer phenotypes in patients with ribosomopathies remain a mystery.

The recent discoveries of somatic mutations in RP genes in hematological cancers (such as T-cell acute lymphoblastic leukemia (ALL), chronic lymphocytic leukemia (CLL) and multiple myeloma) and solid tumors (such as breast cancer and melanoma) emphasize that defects in ribosome biogenesis could potentially promote oncogenic transformation.^23^ In fact, deletions in RP genes are common events across human cancers, particularly in concert with *TP53* mutations. This is in contrast to mutation in RPs (without concurrent *TP53* mutation) that are primarily associated with ribosomopathies.^25^ Here, we review the current knowledge and new perspectives with respect to diseases linked to mutations or deletions in RP genes and the molecular mechanisms driving cancer susceptibility. We highlight how translational rewiring associated with chronic ribosome stress provides mechanistic insights into cancer development and new clinical implications for cancer therapy.

In contrast to RP mutations-associated ribosomal defects in cancers, oncogene-driven cancers are associated with hyperactivation of ribosome biogenesis that promote uncontrolled growth and proliferation and altered metabolism.^26–31^ Indeed, hyperactivation of Pol I transcription of the 47S rRNA precursor and the increase in production of ribosomes are necessary to support the increased rate of protein synthesis required to sustain unrestricted cell growth.^26,32,33^ Intriguingly, many classical chemotherapeutic agents (e.g., oxaliplatin, cisplatin, actinomycin D and 5-fluorouracil (5-FU)) have been discovered to act through distinct mechanisms of action that include inhibition of rRNA synthesis, rRNA processing or ribosome biogenesis^34–38^ (Fig. 1b). The development of a number of less genotoxic drugs that selectively target Pol I transcription and ribosome biogenesis has established a new paradigm for cancer therapy.^36,39–42^ These compounds are active against subsets of tumors but their therapeutic response can be more potent in cancers with deregulated ribosome biogenesis.^43–45^ In this review, we discuss the rationale for targeting ribosome biogenesis as a treatment strategy to combat cancer and the current understanding of the therapeutic potential of the first-in-class inhibitor of Pol I transcription CX-5461. Importantly, recent studies of the key molecular mechanisms of acquired resistance to selective inhibition of ribosome biogenesis provide a new conceptual framework to expand on the understanding that specific rewiring of translation in response to chronic ribosome stress promotes cancer progression. We highlight the potential of this research for the development of novel treatments for human diseases linked to mutations in RPs and deregulated ribosome biogenesis.

## Ribosome biogenesis: a key regulator of cell growth and proliferation

All eukaryotic ribosomes sediment at 80S (S: the Svedberg unit for sedimentation coefficients) and are divided into two distinct subunits of unequal size. The small (40S) subunit functions as a “decoding site,” interacting with the anticodon-containing ends of complementary tRNAs so as to translate the codon information contained in mRNA into its corresponding sequence of amino acids.^46–48^ The large (60S) subunit contains peptidyl transferase activity and is responsible for linking the amino acids into a polypeptide chain.^8,46–48^ Each ribosome subunit forms a ribonucleoprotein particle such that the small 40S subunit of the eukaryotic ribosome contains one 18S rRNA chain and 33 RPs, while the large 60S subunit consists of three rRNA molecules (28S, 5.8S, and 5S) and 48 RPs.^49^

The nucleolus is the largest subnuclear structure, forms around active clusters of the 47S rRNA genes to establish the site of Pol I transcription and is therefore known as the site of ribosome biogenesis. The nucleolar morphological structure is compartmentalized into a fibrillar center (FC), a dense fibrillar component (DFC), and a granular component (GC).^50^ In mammals, the majority of the 47S precursor rRNA is thought to be synthesized at the boundary between the FC and the DFC although a smaller proportion can be transcribed in the FC.^51^ The precursor-rRNA is processed and post-transcriptionally modified in the DFC, where small nucleolar RNAs (snoRNAs) of two different classes of non-coding RNAs catalyze covalent modifications (2‘-*O*-ribose methylation and pseudouridylation) of the rRNA molecules to form the mature 18S, 5.8S, and 28S rRNAs.^8,9,11,51,52^ These mature rRNAs, in conjunction with the 5S rRNA synthesized by RNA Pol III in the nucleoplasm are then assembled in the GC of the nucleolus with RPs encoded by Pol II to form the major catalytic and architectural components of the small (40S) and the large (60S) ribosomal subunits.^11,51,53^ These subunits are then exported to the cytoplasm where they form the functional (80S) ribosome after the final maturation steps.^51,54,55^

Eukaryotic cells have multiple copies of tandemly repeated 47S rRNA genes per haploid genome, termed rDNA repeats.^56,57^ These rDNA clusters of 70–80 repeats are arranged as head-to-tail arrays on the short arms of the five human acrocentric chromosomes.^58–60^ Each repeating rDNA unit possesses the pre-rRNA coding region, encoding the 18S, 5.8S and 28S rRNAs, as well as external (ETS) and internal (ITS) transcribed and non-transcribed regions, separated by intergenic spacer sequences.^13,61^ Although present at high copy number, only a proportion of rDNA repeats are actively transcribed by Pol I at any given time in metabolically active cells.^61–63^ Recent studies indicate that the rDNA repeats are regulated by their distinct chromatin states and epigenetic modifications, and primarily exist in four functional states: (i) silent, (ii) pseudo-silent, (iii) transcriptionally competent but poised, or (iv) transcriptionally active rDNA.^61,64,65^ In general, an open chromatin structure correlates with transcriptional competency and is characterized by DNA hypomethylation, acetylated histones as well as the binding of the upstream binding factor (UBF), which is essential for recruiting Pol I to the rDNA promoter and maintaining the active rDNA state.^62,66^ Inactive rRNA genes, on the other hand, are not bound by UBF and are characterized by repressive histone modifications and CpG hypermethylation at the rDNA promoter and thus are stably silenced, or they can be non-CpG methylated and hence are in a “pseudo-silent” state.^62^ While the production of the 47S pre-rRNA has long been thought to be controlled by modulating the rate of Pol I transcription in response to growth stimuli or cell cycle cues,^16,67–70^ alterations in rDNA chromatin states have also been shown to contribute to the long-term regulation of Pol I transcription such as during cellular differentiation and transformation.^62,71–74^

Dysregulated Pol I transcription is linked to ribosomopathies and cancer. Mutations in factors closely associated with Pol I transcription and deregulation of Pol I transcription are linked to the etiology of a number of ribosomopathies.^21^ In contrast to ribosomopathies, altered Pol I transcription in cancer is largely a result of dysregulated oncogenic pathways upstream of Pol I transcription or due to direct modulation of the Pol I transcription apparatus by oncoproteins or tumor suppressors.^21^ Moreover, due to the repetitive nature and high transcription rate of the rDNA repeats as well as their challenging DNA replication, the rDNA loci are inherently unstable and have been shown to be increasingly susceptible to DNA damage and chromosomal recombination events resulting in large copy number variations.^75,76^ Variation in rDNA copy number is correlated with the expression of functionally coherent genes involved in ribosome biogenesis and this association has been proposed as a mechanism for cellular homeostasis including a rapid and reversible source of adaptation to coordinate ribosome biogenesis.^77^ Genomic instability of the rDNA loci has been reported in congenital diseases characterized as putative ribosomopathies with a high cancer risk, such as Bloom and Werner syndromes.^21,78^

## Canonical and extra-ribosomal functions of RPs

In the context of ribosome assembly and function, the RPs are involved in the stabilization of the small and large subunit structures, rRNA processing and stabilization of secondary structures in the rRNA, pre-ribosome transport, RNA folding and/ or interaction with auxiliary factors required for ribosome assembly and mRNA translation.^79^ This review is focused on the cytoplasmic RPs as opposed to the 75 mitochondrial RPs that assemble into the mitochondrial ribosome. We refer readers to other excellent reviews on mitochondrial RPs and their role in mitochondrial protein synthesis.^80,81^

In addition to their structural and regulatory roles in the assembly of the ribosome, RPs perform other “moonlighting” extra-ribosomal functions including the regulation of cell growth, proliferation and differentiation, immune signaling, DNA repair and apoptosis. These functions are defined based on specific interactions between RPs with non-ribosomal cellular components independent of the ribosome.^82,83^ This review is focused on the extra-ribosomal roles of RPs in development and tumorigenesis. For further reading on RPs extra-ribosomal functions, we refer readers to other reviews.^82–86^

### Nucleolar stress response

**Fig. 2:**
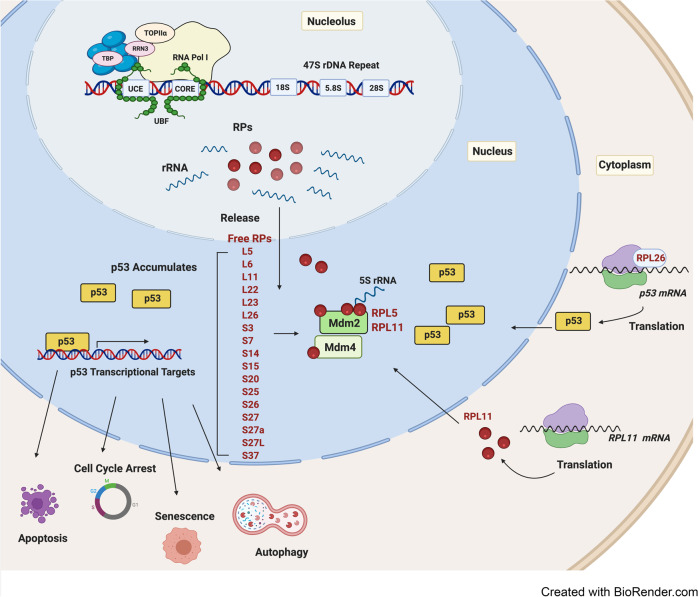
p53-mediated nucleolar stress response. Cell growth and proliferation remain under constant nucleolar surveillance. Under normal growth conditions, levels of the tumor suppressor p53 are suppressed by the binding of the E3 ubiquitin ligase mouse double minute 2 (Mdm2) and its homolog Mdm4, leading to ubiquitination and degradation of p53. When ribosome biogenesis is disrupted at the level of rRNA synthesis, processing or ribosome assembly, free ribosomal proteins (RPs) (primarily RPL5 and RPL11 and RPL23) and the 5S rRNA are released from the nucleolus to the nucleoplasm where they bind and sequester Mdm2/Mdm4. This in turn prevents the poly-ubiquitination and proteasome-mediated degradation of p53, thereby mediating its stabilization. The RPs (indicated) have been shown to regulate the Mdm2/p53 axis through various mechanisms including binding Mdm2 and its homolog and binding partner Mdm4. Additional mechanisms of nucleolar stress response include ribosome stress-mediated increase in *RPL11* mRNA translation, which leads to enhanced interaction between RPL11 and Mdm2 and subsequent accumulation of p53. Following nucleolar stress, p53 can also be activated by RPL26 binding to *p53* mRNA and enhancing its translation. Upon activation, p53 transactivates several downstream targets, leading to cell cycle arrest, apoptosis, autophagy or senescence

In addition to ribosome production, the nucleolus plays a critical role as a central hub in coordinating cellular response to stress by integrating various stress response pathways including activation of the p53 pathway.^84,87^ Central to this activity is the nucleolar stress response (also known as the impaired ribosome biogenesis checkpoint),^28^ whereby perturbations in ribosome biogenesis, such as inactivation of Pol I transcription, impaired rRNA processing, ribosome assembly and transport are established mechanisms of nucleolar stress that induces p53 pathway activation, leading to cell cycle arrest, senescence, autophagy, and apoptosis^87–89^ (Fig. 2).

In response to nucleolar stress, p53 stabilization can be achieved via different mechanisms including post-translational modifications, protein-protein interactions and increases in the translation rate of p53 mRNA. One of the best documented mechanisms of p53 induction upon nucleolar stress involves the binding of RPs to Mdm2 (mouse double minute 2; also referred to as HDM2 in human), leading to p53 stabilization.^90^ In proliferating cells, p53 activity is kept repressed by Mdm2 via two complementary mechanisms: (*i*) Mdm2 acts as an E3 ubiquitin ligase that directly transfers ubiquitin onto p53 thereby targeting it for 26S proteasomal degradation;^91,92^ and (*ii*) the direct binding of Mdm2 to the N-terminal domain of p53 inhibits the transcriptional activity of p53 by preventing its interaction with the Pol II transcription machinery.^93,94^ This Mdm2-dependent surveillance of p53 activity is regulated by distinct and independent mechanisms to those involving replicative stress and the DNA damage response (DDR) where phosphorylation of either Mdm2 or p53 prevents their interaction, leading to p53 stabilization. p53 stabilization by the nucleolar stress response requires RPs.^90,95^

Furthermore, an increasing number of RPs including RPL5,^96^ RPL6,^97^ RPL11,^98,99^ RPL22,^100^ RPL23,^101,102^ RPL26,^103^ RPL37,^104^ RPS3,^105^ RPS7,^106,107^ RPS14,^108^ RPS15,^104^ RPS19,^109^ RPS20,^104^ RPS25,^110^ RPS26,^111^ RPS27,^112^ RPS27A,^113^ and RPS27L^112,114^ have been shown to regulate the Mdm2/ Mdm4 (Mdm2’s homolog and partner also known as MdmX in mice and HDMX in human)/p53 axis, consequently suppressing cell proliferation. A number of different models have been proposed for how these various interactions might regulate p53 (Fig. 2). In the “riding the ribosome” model, the interaction of p53 and/or Mdm2 with ribosomal subunits facilitates p53/Mdm2 transport from the nucleolus to the cytoplasm thereby preventing p53 from activating its target genes and promoting its ubiquitin-mediated degradation in the cytoplasm.^84,86^ Conversely, impairment of ribosome production and export of ribosome subunits is predicted to decrease p53/Mdm2 transport to the cytoplasm, thus allowing p53 to interact with its target genes in the nucleoplasm.^84^ A recently characterized alternative model described that inhibition of rRNA transcription or ribosome synthesis and assembly in the nucleolus creates a pool of free RPs that directly interact and sequester Mdm2. For instance, inhibition of Pol I transcription by a range of treatments including low doses of actinomycin D and oxaliplatin; the reduction of particular RPs; serum starvation; increases in cell confluence and nucleotides depletion^83,86,88,89,115–117^ have all been shown to induce the release of RPL5, RPL11 and RPL23 into the nucleoplasm where they can interact with the central acidic domain of Mdm2, suppressing p53 ubiquitination.^84,86^ Although most of the RPs interact with Mdm2 directly, some of them such as RPS7,^107^ RPS15,^104^ RPS20,^104^ RPS25,^110^ and RPL37^104^ have also been shown to bind to Mdm4. These RPs were shown to employ different mechanisms in regulating the Mdm2-p53-Mdm4 network.^104,107^

RPL5 and RPL11 can associate with each other and 5S rRNA and this pre-ribosomal complex is essential for p53 activation upon impairment of ribosome biogenesis.^118,119^ Intriguingly, depleting RPL5 and RPL11 but not other RPs (e.g., RPS7 and RPL23) is sufficient to reverse cell cycle arrest induced by defects in ribosome biogenesis.^117,120^ However, a synergistic suppression of Mdm2 activity through cooperation of RPL11 and RPL5 has been observed, suggesting they have distinct roles in inhibiting Mdm2 function.^119^ The cancer-associated Mdm2 C305F mutation, which disrupts the interaction between Mdm2 and RPL5 and RPL11 prevents p53 activation in response to nucleolar stress.^121^ A knock-in mouse model with the Mdm2 C305F mutation displayed accelerated Myc-induced lymphomagenesis,^95^ indicating a tumor-suppressive role for the RPL11–RPL5–Mdm2–p53 against tumorigenesis. Mdm2 can also be inhibited by other nucleolar proteins such as p19ARF, nucleophosmin and nucleostemin in response to nucleolar stress.^122,123^

The RPS7/RPL26 association with Mdm2 has a different regulatory function as they are both reported to be substrates for Mdm2 ubiquitination. In turn, an RPS7-ubiquitin fusion protein selectively inhibits Mdm2 degradation of p53 and promotes apoptosis. This indicates that Mdm2 ubiquitination of RPS7 is involved in sustaining the p53 response.^107^ RPL26 is unique in its ability to bind the 5’untranslated region of the *p53* mRNA to promote its translation. Its interaction with Mdm2 triggers its own ubiquitination and degradation, which in turn causes downregulation of *Tp53* mRNA translation^124^ (Fig. 2). Further support for the diverse roles of RPs in the regulation of the Mdm2-p53 pathway stems from the finding that knockdown of RPS6 reduces 40S ribosome biogenesis but increases *RPL11* mRNA translation. This enhances the interaction between RPL11 and Mdm2, leading to p53 activation^125^ (Fig. 2). Since multiple RPs have distinct mechanisms for activating p53, it is plausible that they may sense and integrate different types of signals, leading to activation of nucleolar stress pathways.^90^ Upregulation of p53 as a consequence of defective ribosome biosynthesis and subsequent activation of the nucleolar stress response is linked to a wide spectrum of hypo-proliferative phenotypes displayed by ribosomopathy patients.^18,126,127^

### P53-independent RP-mediated responses to nucleolar stress

In addition to activating p53; RPL5, RPL11 and RPS14 have been shown to bind to the p53 homolog p73 to prevent Mdm2 from binding to p73 at target gene promoters, such as those of p21 and Puma, leading to p73 activation and p73-dependent apoptosis.^128^ Intriguingly, simultaneous knockdown of p73 and either RPL5 or RPL11 was required to rescue 5-FU-induced apoptosis of p53-null tumor cells,^128^ supporting the essential role of RPL5 and RPL11 in p73-mediated apoptosis.

In addition to activating p53 and p73, several RPs can also inactivate oncoproteins, such as c-Myc. RPL11 specifically binds to the Myc box II domain of c-Myc and inhibits its transcriptional activity.^129^ RPL11 was also shown to bind *c-Myc* mRNA and promote its degradation in response to nucleolar stress.^130^ Moreover, both RPL5 and RPL11 co-resided on *c-Myc* mRNA and suppressed c-Myc expression through a RNA-induced silencing complex-mediated miRNA targeting mechanim.^131^ Similarly, RPS14 has also been found to suppress c-Myc transcriptional activity and promote its mRNA turnover.^132^ This reveals a negative feedback regulatory loop of RPs as MYC also activates expression of RPs.^133^ The auto-regulatory feedback regulation of RPs and MYC may act as a sensor of abnormal ribosome biogenesis that consequently limits MYC activity.^133^

Other studies have shown that RPL41 facilitates the shuttling of activating transcription factor 4 (ATF4), a regulator of tumor cell survival, from the nucleus to the cytoplasm to be degraded, consequently sensitizing tumor cells to chemotherapy.^134^ Additionally, RPS3 can induce apoptosis by interacting and collaborating with E2F1, and Akt-mediated phosphorylation of RPS3 attenuates apoptosis by abrogating the RPS3–E2F1 interaction.^135^ Phospho-RPS3 was also shown to shuttle to the nucleus and upregulate pro-survival gene expression via association with NF-κB in non-small cell lung cancer cells.^136^ These studies thus reveal the ribosome-independent functions of RPs and provide further understanding of the extra-ribosomal roles of RPs in tumorigenesis.

## RP mutations and human diseases

Mutations of RP-encoding genes are highly associated with genetic diseases such as ribosomopathies and cancer (Table 1). Large-scale sequencing studies and various experimental models have provided in-depth insights into the ribosomal and extra-ribosomal functions of RPs, which we review in the following sections. In addition, emerging evidence suggests post-translational modifications of RPs, for example, phosphorylation and ubiquitylation, may functionally influence translational control and are linked to human diseases (reviewed in refs. ^137,138^)

**Table 1:** Ribosomal proteins and human disease

Ribosomal protein	Entrez gene ID	Human disease		References
		Developmental disease	Cancers	
RPS7	6201	DBA	Prostate cancer, colorectal cancer, breast cancer, ovarian cancer	^305–309^
RPS10	6204	DBA		^310^
RPS14	6208	Del(5q) MDS		^149^
RPS15	6209	DBA	CLL	^305,311^
RPS15A	6210	DBA	lung, glioblastoma, gastric, liver and colorectal cancer	^217–222,312^
RPS17	6218	DBA	Colorectal cancer	^313,314^
RPS19	6223	DBA	Epidermoid carcinoma	^315,316^
RPS20	6224		Hereditary nonpolyposis colorectal cancer, gastric cancer	^317,318^
RPS23	6228	Brachycephaly, trichomegaly and developmental delay		^319^
RPS24	6229	DBA	Colorectal	^320,321^
RPS26	6231	DBA		^310^
RPS27	6232	DBA	Melanoma	^322,323^
RPS28	6234	DBA		^145^
RPS29	6235	DBA		^324^
RPSA	3291	Isolated congenital asplenia	Pancreatic cancer	^325,326^
RPL3L	6123	Autosomal dominant polycystic kidney disease		^327^
RPL5	6125	DBA	T-ALL, melanoma, multiple myeloma, glioblastoma, breast cancer	^202,305^
RPL9	6133	DBA	Colorectal cancer	^328,329^
RPL10	6134	Autism; X-linked syndromic mental retardation 35	T-ALL, epithelial ovarian cancer	^205,208,330,331^
RPL11	6135	DBA	T-ALL, melanoma, gastric cancer	^202,205,305,332^
RPL13	6137	Spondyloepimetaphyseal dysplasia		^333^
RPL15	6138	DBA	Breast cancer, pancreatic cancer, colon cancer, gastric cancer	^334–338^
RPL18	6141	DBA		^339^
RPL21	6144	Hypotrichosis 12	Pancreatic cancer	^340,341^
RPL22	6146		T-ALL, gastric cancer, endometrial cancer, colorectal cancer, adrenocortical carcinoma	^209–214,342^
RPL23A	6147		Endometrial cancer	^202^
RPL26	6154	DBA		^343^
RPL27	6155	DBA	Nasopharyngeal carcinoma	^322,344^
RPL31	6160		Prostate cancer	^345^
RPL34			Esophageal cancer, Non-small cell lung cancer, esophageal cancer, gastric cancer, pancreatic cancer, glioma cells, osteosarcoma, cervical cancer	^346–352^
RPL35	11224	DBA	neuroblastoma	^339,353^
RPL35A	6165	DBA		^354^
RPL36	25873		Glioma, hepatocellular carcinoma, colorectal cancer	^355–357^

*CLL* chronic lymphocytic leukemia, *T-ALL* T-cell acute lymphoblastic leukemia

### Ribosomopathies

Mutations in RPs or other factors involved in the process of ribosome synthesis and assembly account for the pathogenesis of a heterogeneous group of diseases called ribosomopathies.^15–17^ This review focus on Diamond–Blackfan anemia (DBA) and Myelodysplastic syndrome (MDS) with chromosome 5q deletion (del(5q) MDS) in which RP mutations have been linked to the disease etiology (Table 1), and on diseases associated with mutations in other ribosome biogenesis factors including Shwachman-Diamond syndrome (SDS), X-linked-dyskeratosis congenita (XL-DC), cartilage–hair hypoplasia–anauxetic dysplasia (CHH-AD) and Treacher Collins Syndrome (TCS).

#### Ribosomopathies associated with mutations in RPs

##### Diamond–Blackfan anemia (DBA)

DBA is an autosomal dominant genetic disorder characterized by erythroid aplasia in association with a wide spectrum of congenital anomalies, including craniofacial, limb, genitourinary, and heart malformations, as well as an increased susceptibility to cancer. Since mutations in RPS19 were identified as the first causal genetic lesions for DBA in 1999,^139^ mutations in nineteen of the eighty-one RP-encoding genes have been identified, with RPS19 (25%), RPL5 (7%), RPS26 (6.6%) and RPL11(5%) being the most frequently mutated genes in DBA^140,141^ (Table 2). Approximately 70% of DBA cases are caused by loss-of-function mutations in a single copy of RP genes.^142^ In addition to RPs, several non-RP genes also contribute to the pathogenesis of DBA. Reduced translation of the key erythroid transcription factor GATA1,^143^ increased degradation of the GATA1 chaperone HSP70,^144^ or mutation in the RPS26 chaperone protein TSR2,^145^ impairs erythroid lineage commitment, resulting in specific defects in erythropoiesis in DBA.

**Table 2:** The ribosomal defects, cancer susceptibility and treatment in ribosomopathies

Ribosomopathy	Mutated ribosomal genes (% patients with the disease indicated)	Function in ribosome biogenesis	Clinical features	Cancer susceptibility (O/Z ratio)	Treatment
*Ribosomopathies associated with ribosomal proteins*					
Diamond–Blackfan anemia	RPS19 (25%), large deletion (10-20%), RPL5 (7%), RPS26 (6.6%), RPL11 (5%), RPL35A (3%), RPS10 (3%), RPS24 (2.4%), RPS17 (1%), RPL15, RPS28, RPS29, RPS7, RPS15, RPS27A, RPL9, RPL18, RPL26, RPL27, RPL31, GATA1, HSP70, TSR2	Pre-rRNA processing, ribosomal subunit synthesis and mRNA translation	Bone marrow failure syndrome characterized by erythroid hypoplasia, congenital anomalies including craniofacial, limb, genitourinary and heart malformations	Overall O/Z ratio: 2.5-5.4. MDS (352), vaginal squamous cell carcinoma (172), Esophageal cancer (65), Colon cancer (45), Osteogenic sarcoma (42), AML (29)	steroids; blood transfusion; HSCT
Myelodysplastic syndrome (MDS) with chromosome 5q deletion	RPS14	18S pre-rRNA processing and 40S ribosomal subunit synthesis	Erythroid hypoplasia, macrocytic anemia, hypolobated megakaryocytes with <5% bone marrow myeloblasts, <1% circulating peripheral blasts and absence of Auer rods	MDS, AML	transfusion; erythropoietin; thalidomide; retinoids; chemotherapy and hypomethylating agents, bone marrow transplantation
*Ribosomopathies associated with other ribosome biogenesis factors*					
Shwachman-Diamond syndrome	SBDS (92%), DNAJC21, EFL1	Release of EIF6 from 60S subunit for 60S maturation	Bone marrow failure featured by neutropenia or multilineage cytopenias, multiple developmental anomalies such as exocrine pancreatic dysfunction and impaired bone development	Overall O/Z ratio 8.5. MDS, AML (202), ovarian cancer (169)	Pancreatic enzyme replacement, GCSF, blood transfusions, HSCT; reconstructive surgery
X-linked-dyskeratosis congenita	DKC1(25%)	Pre-rRNA psedouridylation	Bone marrow failure usually associated with skin hyperpigmentation, nail dystrophy, mucosal leukoplakia and pulmonary fibrosis	Overall O/Z ratio 4.2. MDS (578), tongue (216), AML (73), and head and neck tumors (74), leukemia (24)	Androgen, blood transfusion; HSCT;
Cartilage hair hypoplasia–anauxetic dysplasia	RMRP	Pre-rRNA processing	Short stature, bone marrow failure, hair hypoplasia and defective immunity	Overall O/Z ratio 7. Non-Hodgkin lymphoma (100) and basal cell carcinoma (33)	Blood transfusion; reconstructive surgery, HSCT
Treacher Collins Syndrome	TCOF1, POLR1C and POLR1D	Pre-rRNA transcription and modification	Craniofacial malformations	NA	reconstructive surgery

*AML* acute myeloid leukemia, *GCSF* granulocyte colony-stimulating factor, *HSCT* hematopoietic stem cell transplantation, *MDS* myelodysplastic syndrome, *NA* not applicable, *O*/*E* ratio, the observed over expected ratio

##### Myelodysplastic syndrome (MDS) with chromosome 5q deletion (del(5q) MDS)

Del(5q) MDS is a distinct subtype of myelodysplastic syndrome (MDS). The disease is characterized by erythroid hypoplasia, macrocytic anemia, hypolobated megakaryocytes with <5% bone marrow myeloblasts, <1% circulating peripheral blasts and absence of Auer rods as well as the presence of chromosome 5q deletion.^146^ Haploinsufficiency of the genes located in two distinct commonly deleted regions (CDR) of chromosome 5q (5q32 and 5q31) accounts for the pathobiology of this disease.^147^ Among the 40 genes identified in 5q32, haploinsufficiency of RPS14 is a critical molecular event responsible for the erythroid differentiation defect in 5q-syndrome.^148,149^ Alternatively, heterozygous loss of other genes in the CDR including HSPA9 (Heat shock protein family A Hsp70 member 9),^150^ CSNK1A1^151^ encoding for Casein Kinase 1 alpha 1, a component of the beta-catenin complex regulating Wnt/ beta-catenin and p53 signaling pathways, and miR-145 and miR-146a also account for the hematologic features of the Del(5q) MDS.^152^

#### Ribosomopathies associated with mutations in other ribosome biogenesis factors

##### Shwachman-Diamond syndrome (SDS)

SDS is an autosomal recessive disorder associated with bone marrow failure featured by neutropenia or multilineage cytopenias.^153^ It is also characterized by multiple developmental anomalies such as exocrine pancreatic dysfunction and impaired bone development. More than 90% of SDS patients display biallelic inactivating mutations in the SBDS ribosome maturation factor gene (*SBDS*).^154^ SBDS acts in concert with Elongation Factor Like GTPase 1 (EFL1) in removing a ribosomal anti-association factor Eukaryotic Translation Initiation Factor 6 (EIF6) from pre-60S subunits, which is necessary for ribosome maturation.^155–157^ EIF6 is involved in both pre-rRNA processing and export of the pre-60S subunit to the cytoplasm, and thus removal of EIF6 is critical for the association of the nascent 60S subunit with the 40S subunit to form a translationally competent ribosome. In SDS patients, mutation of SBDS stalls 60S maturation and impairs ribosome assembly, resulting in a reduced number of 80S ribosomes.^157^ Recently, the identification of mutations in another 60S ribosome assembly factors DnaJ Heat Protein Family Member C21 (DNAJC21) and EFL1 support that impaired 60S ribosome maturation as a consequence of defective EIF6 eviction is the primary cause of SDS pathogenesis.^158,159^

##### X-linked-Dyskeratosis Congenita (XL-DC)

DC is a genetic disorder characterized by bone marrow failure usually associated with skin hyperpigmentation, nail dystrophy, mucosal leukoplakia and pulmonary fibrosis. The X-linked variant is associated with mutations in *DKC1*, encoding for a pseudouridine synthase Dyskerin.^160^ Dyskerin catalyzes rRNA pseudouridylation by binding to a group of snoRNA containing H-box (ANANNA) and ACA box (ACA) sequence motifs in rRNA, a process important for ribosome production and function. In addition to rRNA modification, Dyskerin also bind to H/ACA sequence in the nascent telomerase RNA component (TERC). The role of Dyskerin in the regulation of telomere function is in line with other gene mutations identified in DC such as TERC and TERT (telomerase reverse transcriptase).^161^ Thus, telomere dysfunction rather than ribosomal defects has been considered to directly contribute to pathogenesis of DC.

##### Cartilage hair hypoplasia–anauxetic dysplasia (CHH-AD)

CHH-AD is an autosomal recessive disease presented with short stature, bone marrow failure, hair hypoplasia and immunodeficiency. It is caused by mutations in RMRP (RNA Component of Mitochondrial RNA Processing Endoribonuclease), a long non-coding RNA component of the ribonuclease MRP complex involved in pre-rRNA processing, to give rise to the mature 18S and 5.8S rRNAs.^162^ In addition to its role in ribosome biogenesis, the MRP complex has pleotropic effects on the processing of mitochondrial RNA and cell cycle-associated mRNAs.

##### Treacher Collins Syndrome (TCS)

Distinct from DBA, SDS, X-linked DC, and CHH-AD, which are classified as inherited bone marrow failure syndrome, TCS is a rare congenital disorder characterized by various craniofacial malformations. It is primarily associated with autosomal dominant mutations in *TCOF1*, a gene encoding for a 144 kDa protein called Treacle.^163^ Treacle is responsible for pre-rRNA transcription and methylation.^164,165^ Furthermore, defects in pre-rRNA synthesis by TCOF1 depletion are associated with the delocalization of the nucleolar DExD-Box Helicase 21 (DDX21), a DEAD box RNA helicase involved in rRNA synthesis and processing, to the nucleoplasm, leading to inhibition of ribosome biogenesis.^166^ In addition to its function in rRNA synthesis and modification, a recent study revealed a critical role for TCOF1 in DNA repair by the formation of a component of the MDC1-RAD50-NBS1-MRE11 complex. Haploinsufficiency of *Tcof1* perturbs the DNA damage response and causes the extensive apoptosis of neuroepithelial cells associated with the pathogenesis of TCS.^167^ In addition to *TCOF1*, mutations in *POLR1C* and *POLR1D*, which encode subunits of both RNA polymerase I and III, are also associated with TCS.^168,169^ Like *TCOF1* mutations, decreased pre-rRNA transcription and number of functional 80S ribosomes were found in both *polr1c*- and *polr1d*-mutant zebrafish models of TCS.^170,171^ These findings highlight a causal link between ribosomal defects and the pathogenesis of TCS.

The extent to which ribosomal defects in ribosomopathies contribute to clinical phenotypes remains to be defined. As discussed above, in TCS, DBA and SDS, the defects in ribosome production and function have been causally linked to clinical phenotypes. However, in other putative ribosomopathies such as XL-DC and CHH-AD, it is likely that the ribosomal defects contribute to a component of their disease features and impact on disease severity.^20^

A prominent characteristic of ribosomopathies is tissue-specific defects. Despite the ubiquitous requirement for ribosomes in all cell types, patients frequently exhibit symptoms arising from tissue-specific growth arrest, including bone marrow failure, anemia or other tissue developmental defects.^172^ Nevertheless, these diseases collectively possess many overlapping clinical features. For instance, the patients commonly present with symptoms of aging including deafness, cataracts and loss of subcutaneous fat. Patients with DBA and SDS have decreased numbers of hematopoietic stem cells (HSCs), with a significant subpopulation of their HSCs showing senescence markers including G1/S cell cycle arrest, SA-β-galactosidase and γH2AX staining, suggesting that ribosomopathies are associated with premature senescence.^173^ As disruption of ribosome biogenesis at various steps can promote cell cycle arrest and senescence, cellular senescence may underpin the common defects observed in ribosomopathies. A detailed description of the molecular mechanism underpinning ribosomopathy phenotypes is presented below. While senescence is an early feature of ribosomopathies, in the long-term, cancer susceptibility has been observed in patients with ribosomopathies except TCS. In this review, we also summarize and discuss the current understanding of the mechanisms underpinning an increased cancer risk in ribosomopathies.

#### The molecular basis of ribosomopathies

##### The nucleolar stress response

A critical role for p53 activation in the pathogenesis of ribosomopathies has been well established in zebrafish, murine or human disease models, including DBA,^174–176^ del (5q) MDS,^148^ SDS,^153^TCS^177^, and XL-DC.^178^ The nucleolar stress response triggered by ribosomal defects is the primary cause of p53 stabilization and activation. In DBA, RP haploinsufficiency results in impaired rRNA processing and disruption of the cognate 40S or 60S subunit biosynthesis,^179^ leading to accumulation of free RPs, including RPS3, RPS7, RPL5, RPL11, and RPL23 in the nucleoplasm. As noted above RPL5 and RPL11 together with 5S rRNA play a central role in the activation of the p53-dependent nucleolar stress signaling pathway^180^ (Fig. 2). In TCS, apoptotic elimination of neuroepithelial cells and neural crest cells upon p53 activation mediated by the nucleolar stress response has been considered to be the primary cause of craniofacial anomalies in TCS.^177^ Genetical or pharmacological inactivation of p53 can rescue disease-associated phenotypes, strongly supporting that p53 is a key molecular mediator of the hypo-proliferative clinical symptoms of ribosomopathies.^18,19,126,127^

##### Altered mRNA translation by ribosomal defects

**Fig. 3:**
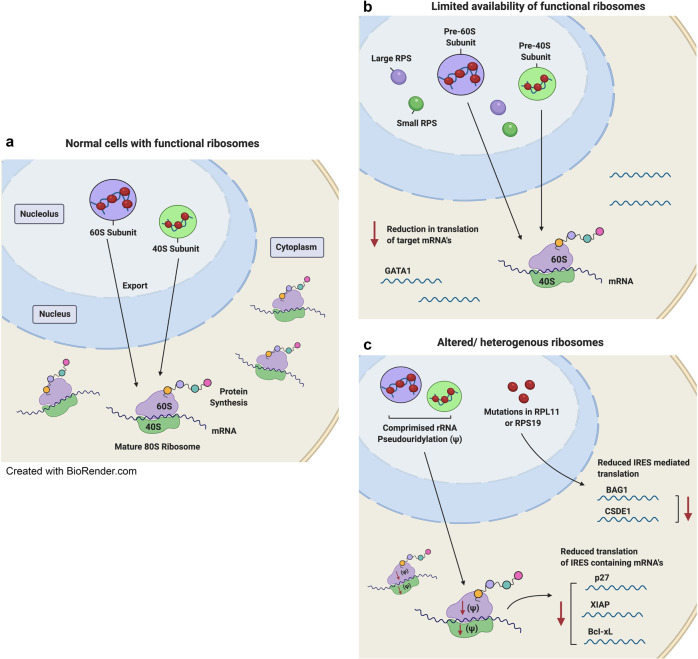
Altered mRNA translation in cells with ribosome biogenesis defects. **a** In normal cells, functional mature ribosomes (80S) comprise the small (40S) subunit and the large (60S) subunit. The small subunit interacts with the anticodon-containing ends of complementary tRNAs so as to translate the codon information contained in mRNA into its corresponding sequence of amino acids. The large subunit contains peptidyl transferase activity and is responsible for linking the amino acids into a polypeptide chain. **b** In ribosomopathies such as DBA (Diamond–Blackfan anemia), mutations in RPS19 can cause a decrease in the number of functional ribosomes, which may lead to a competition for ribosomes among cellular mRNAs, leading to changes in the translation efficiency of subsets of mRNAs, including reduced translation of *GATA1* mRNA. Reduced levels of GATA1, a key erythroid transcription factor impairs erythroid lineage commitment and results in specific defects in erythropoiesis in DBA. **c** Ribosome defects due to RP mutations and variation in RP composition may generate heterogeneous ribosomes with reduced translation fidelity, resulting in altered translation patterns. In DBA patients, deficiencies in RPL11 or RPS19 due to mutations can reduce the translation of IRES-containing mRNAs *BAG1* and *CSDE1*, which encode erythroblast proliferation and differentiation factors. In X-linked-Dyskeratosis Congenita, defects in rRNA pseudouridylation can impair the binding of ribosomes to IRES elements, resulting in reduced translational fidelity and decreased translation of several IRES-containing mRNAs, including *p27*, *XIAP*, and *Bcl-xL* and enhanced bone marrow failure and cancer susceptibility

As ribosomes are essential for translating mRNA into proteins, defects in ribosome synthesis and function alter the translation capacity and efficiency of ribosomes (Fig. 3). Genome-wide translational profiling of cellular models of ribosomopathies has revealed that translation of subsets of mRNAs, particularly mRNAs encoding proteins involved in cell fate decisions, are specifically impaired under the limited availability of functional ribosomes.^23^ Particularly, the specific reduction in translation of *GATA1*, a master regulator of hematopoiesis, contributes to bone marrow erythroid hypoplasia in DBA patients.^143,181,182^ Selective translation defects have also been described in some mRNAs containing an internal ribosome entry site (IRES). Deficiencies in RPL11 or RPS19 reduced IRES-mediated translation of the erythroblast proliferation and differentiation factors *BAG1* and *CSDE1* in DBA murine models and patient samples.^183^ In addition, rRNA pseudouridylation defects in XL-DC caused a defect in ribosome binding to IRES elements, resulting in reduced translational fidelity and decreased translation of several IRES-containing mRNAs, including the tumor suppressor p27 and the antiapoptotic factors XIAP and Bcl-xL. These factors are linked to two specific pathological features of XL-DC: susceptibility to cancer and bone marrow failure.^184^

The altered translatome is thought to contribute to the tissue specificity of ribosomopathies. Two hypotheses have been proposed including the “altered/heterogenous ribosome” hypothesis and the “ribosome concentration” hypothesis to explain the selective mRNA translation in determination of disease pathogenesis^19^ (Fig. 3). The altered/ heterogenous ribosome hypothesis argues that variations in rRNA sequence, RP composition or ribosome-associated proteins can result in heterogeneous ribosomes with differential interactions with mRNAs, resulting in the preferential translation of particular mRNAs.^185^ On the other hand, the ribosome concentration hypothesis argues that a limited number of translationally competent ribosomes may cause competition for ribosomes among cellular mRNAs, leading to changes in the translation efficiency of subsets of mRNAs.^182^ mRNAs with long, highly structured 5’UTRs are speculated to be more affected by an insufficiency of ribosomes. This is supported by a study showing that reduction of *GATA1* mRNA translation in DBA patients is attributed to its highly structured long 5’UTR.^143^ Paradoxically, recent reports have shown that reduction of ribosome abundance impairs the translation of mRNAs that are normally highly translated and have short/unstructured 5’UTRs.^181,182^ These contradictory results indicate that further defining the effects of structural features of mRNAs on translation efficiency will improve our understanding of translation specificity regulated by ribosome availability and their contribution to the pathological features of ribosomopathies.

While p53 activation and mRNA translation reprogramming as a result of impaired ribosome synthesis and function contribute to the disease phenotypes associated with RP haploinsufficiency, these two mechanisms have long been considered to be mutually exclusive in ribosomopathies. Intriguingly, a recent study of a DBA embryonic mouse model has demonstrated that *Rps6* haploinsufficiency leads to selective limb phenotypes, mediated by deregulation of translation. However, the majority of mRNAs undergoing differential translational changes were rescued upon the loss of p53,^186^ indicating an intimate link between translational control and p53 activation upon ribosome perturbation in ribosomopathies. They further demonstrated that p53 transcriptionally induces the expression of 4E-BP1, a negative regulator of eIF4E-mediated cap-dependent translation and that the p53-4E-BP1-eIF4E axis contribute to the selective changes in cap-dependent translation.^186^ Therefore, an altered translatome within specific cellular and/or tissue contexts in response to p53 activation may contribute, in part, to the tissue-specific phenotypes of ribosomopathies.

##### Oxidative stress and deregulated protein degradation

It is becoming clear that fully functional ribosomes are required for cellular redox homeostasis. Increased reactive oxygen species (ROS) levels were detected in murine DBA cells with RPL5 and RPS19 deficiency^187^ and in lymphocytes from DC patients.^188^ Reduction of ROS levels by antioxidant treatment rescued the growth defect in cells with RPL10 mutation or with SBDS inactivation, reinforcing a strong link between oxidative stress and ribosomal defects.^189^

Excessive ROS causes oxidative damage to all macromolecules, including nucleic acid, proteins, and lipids. The high abundance of rRNA and RPs render them susceptible to chemical modification by ROS, leading to further disruptions in ribosome assembly and function.^190,191^ Indeed, *Tcof1*^*+/−*^ embryos exhibited a high level of ROS in the neuroepithelium and the impaired DNA repair capacity resulted in increased sensitivity of neuroepithelial cells to oxidative stress and extensive apoptosis.^192^ Oxidative stress also causes mitochondrial dysfunction and suppresses oxidative phosphorylation and ATP production, which contributes to the hypo-proliferative phenotype observed in ribosomopathies.^193^

While the significance of oxidative stress in the pathogenesis of ribosomopathies is increasingly being recognized, the mechanisms by which defective ribosome biogenesis causes ROS accumulation remain unclear. Two recent studies suggested that selective defects in *Globin* mRNA translation lead to the imbalance of heme-globin, resulting in excess free heme, which contributes to ROS production followed by apoptosis and delayed erythroid differentiation in DBA patients.^194^ In addition, p53 activation in the mesenchymal cells of the hematopoietic niche with *Sbds* deletion led to secretion of the inflammatory molecule S100 A8/A9, which subsequently induced oxidative stress and the DNA damage response in the surrounding wild-type hematopoietic stem and progenitor cells, contributing to impaired hematopoiesis in SDS.^195^ Therefore, the oxidative stress that occurs in ribosomopathies is a downstream effect of either intracellular ribosomal defects or microenvironmental assaults.

While ribosomes function to synthesize proteins, recent studies suggest a potential interaction between ribosomes and the protein degradation machinery in maintaining protein homeostasis.^196^ A biochemical analysis of RPS19 variants revealed that a subset of mutant RPS19 proteins failed to localize to the nucleolus and exhibited a dramatically reduced level of protein expression. Proteasome inhibitors effectively restored mutant RPS19 protein expression levels and nucleolar localization.^196^ Reduced stability of mutant RPs via proteasomal degradation was also observed in the cells expressing mutant RPS15 proteins.^197^ These findings thus underscore a proteasome-based mechanism of quality control of mutant RPs.

Conversely, RP mutations have been demonstrated to alter proteasomal activity. Cells with an RPL10-R98S mutation exhibit aberrant expression of several proteasomal proteins including upregulation of Psmb10 and Psmb9, the catalytic subunits for the immunoproteasome, along with reduced chymotrypsin-like and caspase-like activities of the proteasome.^198^ The altered expression and activities of proteasomes in RPL10- R98S expressing cells led to an elevated sensitivity to proteasome inhibitors. Targeting the proteasome is also proposed to be a novel therapeutic approach for treatment of TCS. Degradation of the cellular nucleic acid-binding protein (Cnbp), a protein involved in craniofacial development, was identified to antagonize the TCS phenotype in a zebrafish model of TCS. Proteasome inhibitors ameliorate cranial skeleton anomalies exhibited in TCS-like embryos by decreasing degradation of Cnbp, strengthening a critical role of the functional ribosomes in cell proteostasis.^199^

### Cancer

#### Cancer susceptibility in ribosomopathies

Patients with ribosomopathies have an elevated risk of developing cancer throughout their life, despite the hypo-proliferative phenotypes associated with early symptoms, and for particular cancer types the risk can be up to 200-fold higher^23^ (Table 2). The cancer susceptibility of DBA has been recapitulated in the mouse model with heterozygous deletion of *Rpl11.*^200^ The paradoxical transition from an early hypo-proliferative cellular response to the hyper-proliferation oncogenic phenotype later in life, was first reported by Dameshek in 1967 and referred to as Dameshek’s riddle.^24^ The recent discoveries of somatic mutations in RP genes in hematological cancers and solid tumors reinforce the link between defects in ribosome biogenesis and oncogenic transformation.^17,19,20^ Here, we describe a range of common cancer-associated mutations mainly reported in RPL5, RPL11, RPL10, RPL22, RPS15 and RPS15A occurring in overlapping and distinct cancers (Table 1) and we discuss the current understanding of the mechanisms of oncogenesis of RP mutations including impaired nucleolar stress response and altered mRNA translation.

#### Somatic RP mutations in cancer

##### RPL5 and RPL11

As mentioned above, RPL5 and RPL11 mediate activation of the nucleolar stress response by inhibiting Mdm2 leading to p53 induction. Heterozygous RPL5 and RPL11 mutations or deletions have been identified in spontaneous human cancer. A comprehensive analysis of TCGA/ICGC pan-cancer dataset including 19,000 cancer samples across 49 cancer types detected 139 RPL5 and 74 RPL11 cancer-associated mutations, of which the majority are missense mutations (66% and 73% in RPL5 and RPL11 mutations, respectively).^201^ Various frequencies of RPL5 mutations/deletions have been reported in breast cancer (34%), melanoma (28%), multiple myeloma (up to 30%) and T-ALL (2%).^202^ In multiple myeloma, patients with low RPL5 expression level have a worse survival outcome. However, analysis of clinical trial data has revealed that multiple myeloma patients with low *RPL5* mRNA expression levels respond better to the proteasome inhibitor bortezomib by comparison with patients with high RPL5 expression,^203^ highlighting the link described above between RP mutations and altered proteasomal activity.

##### RPL10

Somatic mutations in RPL10 at residues R98 and Q123, including R98S, R98C, and Q123P are thought to play an active role in tumorigenesis. Particularly, R98S has been described as a mutation hotspot with >90% of RPL10 mutations at this residue, accounting for ~8% of pediatric T-ALL.^204,205^ The mutants including I33V, E66G, I70M and I70L in RPL10 have been reported in multiple myeloma with low frequency (2%) and cluster in a region that is distinct from the mutation hotspot identified in T-ALL.^206^ An increase in RPL10 expression has been observed in ovarian and pancreatic cancers and linked to enhanced cell proliferation, invasion, survival, and resistance to oxidative stress.^207,208^

##### RPL22

Inactivation of RPL22 due to heterozygous deletion has been observed in 10% T-ALL patient samples. Rpl22 haploinsufficiency accelerated the development of thymic lymphoma in a mouse model of T-cell lymphoma driven by hyperactivation of AKT2 in T-cell progenitors, supporting a tumor-suppressive role for RPL22 in T-ALL.^209^ Homozygous deletion of Rpl22 resulted in distinct phenotypic characteristics with expanded thymic tumors at the primary site in the absence of dissemination and migration to peripheral organs, suggesting a genetic dosage-dependent model in which loss of a single allele of Rpl22 promotes lymphomagenesis and cancer dissemination, while loss of both copies impairs migration capacity and restricts malignant cells to the thymus.^210^ RPL22 mutations have also been described in ∼10% of gastric, endometrial, and colorectal and adrenocortical solid cancer samples.^211–214^

##### RPS15 ad RPS15A

RPS15 mutations have been reported in 19.5% of aggressive CLL and in 12% in CLL with 17p deletion that is typically refractory to most conventional therapies.^215^ The RPS15 heterozygous S138F mutation drove CLL-like disease development in mice and combination with heterozygous *Trp53* deletion shortened the latency of CLL and generated a more aggressive disease course.^216^ In contrast to the tumor-suppressive role of RPS15, RPS15A has been reported to promote oncogenic transformation and progression in lung,^217^ glioblastoma,^218,219^ gastric,^220^ liver^221^ and colorectal cancer.^222^ A decrease in RPS15A expression inhibited proliferation of human glioblastoma cells and gastric cancer cells via downregulation of AKT pathway acitvity,^218,220^ and colorectal cancer cells via activation of the p53 pathway,^222^ and impaired angiogenesis in hepatocellular carcinoma by increasing FGF18 expression via the Wnt/Beta-Catenin pathway.^221^

#### Mechanisms of oncogenic potential RPs

Extensive research of cancer susceptibility in patients with ribosomopathies and somatic RP mutations in cancer has reveal an oncogenic potential of ribosomes. Three mechanisms for formation of “onco-ribosomes” have been proposed, including: *(i)* a shift in translational profiles towards synthesis of growth-promoting and pro-oncogenic proteins; *(ii)* extra-ribosomal functions of RPs beyond ribosome synthesis contributing to oncogenic transformation; and *(iii)* ribosomal defects-associated metabolic alterations promoting secondary mutations and genomic instability. These mechanistic insights not only assist in resolving Dameshek’s riddle, but also facilitate identifying therapeutic vulnerabilities and novel treatments for patients with ribosomopathies and cancer.

##### Translational reprogramming

While the DBA-associated germline mutations in RP genes are usually different from the cancer-associated somatic mutations, with only several variants in common,^223^ it is proposed that mutations in RPs can lead to common effects in deregulating ribosome synthesis and function by either reducing the number of competent ribosomes or forming heterogenous ribosomes (Fig. 3). These ribosomal defects lead to altered translational fidelity and capacity. The evidence for ribosomal defects-induced proteomic changes in the pathogenesis of ribosomopathies has been discussed above. Similarly, structural analysis of somatic cancer-associated RP mutations, including the mutation hotspots in RPL10 and RPS15 revealed that these RP mutations are localized in the regions associated with the catalytic core of ribosome, consistent with their influence on mRNA translation and thus global protein synthesis.^197,224^

An altered translation pattern in cancer cells with RP mutations may promote oncogenic protein synthesis. For instance, the T-ALL-associated RPL10-R98S mutation protects leukemia cell survival via specific upregulation of IRES-dependent translation of the antiapoptotic molecule BCL2.^198^ Studies of cancer susceptibility in ribosomopathies also provide the evidence for oncogenic transformation through translation reprogramming. In SDS, SBDS mutations specifically affect translation of the C/EBPα and β proteins, which are important regulators of hematopoietic granulocyte differentiation. The inability to translate C/EBP α and β is associated with impaired hematopoiesis in SBDS, and more importantly, the 200-fold elevated risk of developing AML in SDS patients,^225^ as loss-of-function mutations in C/EBPα have a known role in AML pathogenesis.^226^ The current findings support the hypothesis that altered translational capacity and fidelity resulting from defective ribosomes contribute to cancer development by facilitating the production of oncoproteins and/or activation of oncogenic pathways.

##### Impaired nucleolar stress response and pro-oncogenic extra-ribosomal functions of RPs

The well-characterized nucleolar stress response whereby the 5S-RNP complex formed by RPL5, RPL11 and 5S rRNA mediates regulation of p53 activation is considered to be an important barrier to cancer development upon defects in ribosome biogenesis. RPL5 or RPL11 loss-of-function disrupts p53 activation and consequently contributes to the propensity of DBA patients to develop cancer. Studies in Rpl11 heterozygous mice showed increased susceptibility to radiation-induced lymphomagenesis. In this regard, total or partial deletion of Rpl11 compromised p53 activation upon ribosome stress or DNA damage in fibroblasts.^200^ RPL5 mutations in human cancer cell lines impair p53 activation, providing supporting data for RPL5/RPL11 haploinsufficiency in promoting malignant transformation via an impaired nucleolar stress response.^200,201^ In agreement with this, the nucleolar proteins SPIN1 and PICT1, which sequester RPL5 and RPL11, respectively in the nucleolus impair the Mdm2-p53 pathway and promote tumor growth by preventing RPL5 and RPL11 from interacting with Mdm2 in the nucleoplasm.^227,228^

Moreover, the extra-ribosomal functions of RPs may confer pro-oncogenic capacity. As noted above, RPL11,^229^ RPL5^131^ and RPS14^132^ can repress MYC expression by either binding directly to the promoter region and reducing transcription or accelerating mRNA degradation. Thus, the loss of these RPs may promote transformation by oncogenic c-MYC overexpression. Indeed, c-MYC upregulation has been described in lymphoma models of heterozygous *Rpl11* or *Rpl22.*^200,209^

##### Metabolic alterations and oxidative stress

The metabolic alterations-associated with ribosome defects also contribute to the oncogenic potential of RPs. The T-ALL-associated RPL10-R98S mutation has been linked to oxidative stress,^189^ which potentially increases genomic instability and places selective pressure towards acquire rescuing mutations, ultimately leading to oncogenic transformation. Metabolic changes can occur though transcriptional, translational and post-translational modulation. The upregulation of phosphoserine phosphatase (PSPH) transcription and translation detected in the lymphoid cells with the RPL10-R98S mutation was thought to contribute to ribosomal mutation-driven serine/glycine synthesis in T-ALL.^230^ In addition, altered proteasome activity in RP mutant cells induces proteomic changes and promotes pro-oncogenic pathway activation. A decrease in Jak1 degradation was found in RPL10-R98S mutant cells and led to activation of the JAK-STAT signaling pathway, an established oncogenic driver in T-ALL.^198^ These findings thus highlight the critical role of metabolic reprogramming in RP mutation-driven oncogenesis.

#### Dysregulation of Pol I transcription of 47S rRNA genes in cancer

In contrast to cancers associated with RP mutations and deletions, oncogene-driven cancers are linked to hyperactive Pol I transcription.^21^ Moreover, the rDNA loci are inherently unstable and susceptible to DNA damage and chromosomal recombination events resulting in large copy number variations.^75,76,231^ However, increased rRNA synthesis can be achieved even if rDNA copy number is reduced,^232^ which can occur by increasing the rate of Pol I transcription per rDNA repeat and/or the number of active rDNA repeats. Furthermore, variation in rDNA copy number independent of Pol I transcription rate has been associated with cancer.^233^ Deregulation of rDNA silencing and/or increased rDNA instability have been proposed to promote global genomic instability and tumorigenesis.^231,232,234–236^

For many decades, an increase in nucleolar size and number, indicative of high rates of Pol I transcription and ribosome biogenesis, has been used as a biomarker of poor cancer prognosis.^32,237^ Indeed, recent studies have confirmed that hyperactivation of Pol I transcription of rRNA genes is a key step in malignant transformation.^39,41,238^ The upregulation of Pol I transcription rates observed in cancer is largely mediated through the deregulation of upstream oncogenic and tumor-suppressive signaling pathways known to modulate ribosome biogenesis, including RAS/RAF/ERK, PI3K/AKT/mTOR, c-MYC, p53, pRb, and PTEN.^31,45,133,239–243^ These oncogenic growth-promoting pathways converge to directly control key players of Pol I transcription initiation such as RRN3 and UBF, leading to enhanced Pol I transcription and cellular transformation.^15,244^ For example, c‐MYC, a potent transcriptional driver of ribosome biogenesis, including the synthesis of rRNAs and RPs,^240,245–250^ is dysregulated or amplified in greater than 70% of all cancers.^251^ RAS and RAF are also mutated in 30% and 6–7% of human cancers, respectively.^252,253^ Activating mutations in the *PIK3CA* gene or inactivating mutations in the negative regulator PTEN are drivers of ~30% of human sporadic tumors.^254,255^ These oncogenic networks cooperate to enhance rRNA synthesis, ribosome biogenesis and protein synthesis at multiple levels and promote “translation addiction” in cancer cells. Thus, targeting ribosome biogenesis has emerged as a potential therapeutic approach to combat cancer.^41,256^

## Therapies targeting ribosomal defects

### Therapies for ribosomopathies

The current standard of care for ribosomopathies encompasses management of the symptoms associated with tissue-specific phenotypes such as bone marrow failure and anemia. This includes chronic blood transfusions followed by steroids to allow patients to survive this disease phase (Table 2), but with many undesirable and even fatal side effects after long-term of steroids, including hypertension, diabetes mellitus, and growth retardation.^257^ There is clearly an unmet need in developing new therapeutic strategies for ribosomopathies.

Here, we summarize the recent research of prospective therapeutics for DBA and del(5q) MDS, the two ribosomopathies related to RP mutations. DBA patients are treated with transfusions followed by corticosteroids. Patients who stop responding to steroid treatment but are transfusion-dependent are then treated with hematopoietic stem cell transplantation (HSCT), which requires an HLA-matched donor and a lifelong immunosuppressive therapy. Thus, the implementation of alternative therapies for the cure of DBA warrants investigation. A range of new compounds have been tested for DBA treatment. The amino acid Leucine improves anemia and developmental defects in DBA zebrafish and mouse models and patients.^258,259^ l-leucine stimulates mRNA translation through activation of the mTOR (the mammalian target of rapamycin) pathway, which regulates cap-dependent translation of TOP mRNAs to synthesize proteins involved in the translational apparatus.^259,260^ The efficacy of l-leucine for the treatment of transfusion-dependent DBA is being evaluated in Phase I/II clinical trials (NCT01362595, NCT02386267, https://clinicaltrials.gov/ct2/home). A recent finding showing that p53 induces the expression of the translation inhibitor 4E-BP1, a negative regulator of eIF4E-mediated cap-dependent translation upon RP haploinsufficiency in a murine model of DBA^261^ also strongly supports targeting cap-dependent translation as a promising approach to treating ribosomopathies. Furthermore, the loss of p53 and thus the ability to repress eIF4E-mediated translation could be a mechanism of the increased cancer risk associated with DBA.^261^ Indeed, increased eIF4E activity has been shown to promote cellular transformation.^262–264^ Moreover, RP genes are routinely deleted across human cancers, particularly in concert with *TP53* mutations.^25^ Therefore, inhibitors of cap-dependent translation such as rapamycin and other inhibitors of mTOR may also serve as potential candidates to treat cancers characterized by RP gene deletions and loss of p53.

In addition to l-leucine, there are three drugs currently being evaluated in clinical trials for treatment of bone marrow failures in DBA: Sotatercept (NCT01464164), TFP (NCT03966053) and EPAG (NCT04269889). Sotatercept is a recombinant human fusion protein containing the extracellular domain of activin receptor type IIA, which inhibits TGF-β signaling. Its murine analog RAP-011 improved erythropoiesis in a DBA zebrafish model.^265^ Trifluoperazine (TFP), a calmodulin inhibitor, rescued the anemia phenotype in different DBA zebrafish and murine models by decreasing p53 translation and accumulation.^266^ Eltrombopag (EPAG), a thrombopoietin (TPO) receptor agonist, increased hemoglobin and platelet numbers in one case,^267^ and rescued erythroid maturation defects in a cellular model of DBA.^268^ In addition, recent studies utilizing gene therapy gene to deliver wild-type RPS19 in lymphoblastoid cell lines (LCLs) established from RPS19-deficient DBA patients rescued impaired ribosome synthesis and reduced p53 levels in LCLs.^269^ A similar approach also rescued the anemia and bone marrow failure phenotype in RPS19-deficient DBA murine model, demonstrating the in vivo efficacy of gene therapy and its potential for curing genetic disorders in ribosomopathies.^174,270^

MDS with del(5q) is managed with red blood cell transfusion (RBC) or recombinant erythropoietin, thalidomide, and retinoid injections to treat the primary symptoms of chronic anemia and fatigue. The majority of patients become RBC-dependent and long-term blood transfusion have multiple adverse effects, including iron accumulation, myocardial infarction, renal failure, infection and malignancy, resulting in increased morbidity, and mortality. Lenalidomide, an immunomodulatory agent has been approved for the treatment of transfusion-dependent anemia in del(5q) MDS with prognostic implication defined as low-risk according to the International Prognostic Score System (IPSS) and the revised IPSS (IPSS-R).^271^ The cytogenetic responses were assessed in a phase II study (MDS-003) involving 148 transfusion-dependent del(5q) MDS patients^272^ with 73% responding to lenalidomide treatment at 10 mg daily and 45% achieving a complete cytogenetic remission, and a phase III study (MDS-004) involving 204 patients with 50% responding in the 10mg- cohorts.^273^ Current clinical data indicates that lenalidomide treatment significantly improved overall survival in patients with low-risk MDS with and without del(5q).^274^ Mechanistically, lenalidomide selectively suppresses MDS clones in patients with del(5q) by targeting the haploinsufficient genes in the CDR of chromosome 5q and the associated pathways. The *Csnk1a1* gene that resides within the CDR for del(5q) MDS encodes for Casein kinase 1a1 (CK1a). In a hematopoietic-specific conditional knockout murine model, heterozygous loss of *Csnk1a1* causes β-catenin accumulation and stem cell expansion while homozygous loss of *Csnk1a1* cause cell apoptosis via of p53 activation.^151^ Lenalidomide induced ubiquitination and degradation of CK1a in heterozygous *Csnk1a1* cells, leading to p53 induction and apoptosis by binding to Cereblon (CRBN), the substrate adaptor for a CUL4A-RBX1-DDB1 E3 ubiquitin ligase complex and modulating the substrate specificity of the enzyme.^275^ Therefore, the synthetic lethality between lenalidomide and *Csnk1a1* haploinsufficiency in chromosome 5q deletion may underpin the clinical efficacy of lenalidomide in del(5q) MDS. In line with this, *TP53* mutations have been linked to the lenalidomide resistance in del(5q) MDS,^276,277^ confirming the primary mechanism of action of lenalidomide is via p53 activation. Indeed, almost 40% of patients progress to acute leukemia by 5 years after starting lenalidomide treatment, most commonly due to clonal evolution associated with acquiring mutations in in *TP53*, *RUNX1*, and *TET2.*^278^

### Targeting ribosome biogenesis as a therapeutic approach in oncogene-driven cancer

Inhibiting oncogene-driven upregulation of ribosome biogenesis in cancer provides a therapeutic specificity for selectively targeting cancer cells over normal cells.^13,41,256,279^ Classical chemotherapeutics (e.g., oxaliplatin, cisplatin, actinomycin D and 5-FU) and poly-ADP ribose polymerase (PARP) inhibitors, recently FDA approved for ovarian cancer treatment, have been discovered to act through distinct mechanisms of action that include inhibition of rRNA synthesis, rRNA processing or ribosome biogenesis^34,35,37,38,280,281^ (Fig. 1b). Indeed, many cancer therapies originally intended to cause DNA damage and kill cancer cells actually impair ribosome biogenesis via multiple mechanisms subsequently leading to cell cycle arrest and senescence.^36,280^

A drug screen by Burger et al., has shown that 21 of 36 chemotherapeutic agents affect ribosome biogenesis at the level of rDNA transcription or rRNA processing.^34^ For example, actinomycin D, which intercalates GC-rich regions of DNA, selectively targets rDNA at concentrations as low as 5 nM and prevents Pol I transcription elongation.^282^ The anti-metabolite 5-FU, an inhibitor of nucleotide synthesis via depleting the intracellular deoxynucleotide pool,^283^ also disrupts rRNA processing.^35^ In addition, the platinum-containing compound cisplatin known to act as a DNA cross-linker inhibits Pol I transcription with a high degree of specificity^284^ through its ability to cross-link DNA at HMG-protein affinity sites, thus preventing the transcription factor UBF from associating with rDNA promoters.^38,285^ Moreover, oxaliplatin, another platinum compound used in the treatment of colorectal cancer, was also shown to kill cells by inhibiting Pol I transcription and inducing ribosome biogenesis stress but not by activating the DNA damage response.^280^ These observations provide the rationale for selectively targeting the Pol I transcription apparatus as a new class of anticancer therapies with reduced toxicity and improve efficacy compared to conventional genotoxic chemotherapeutic agents. Indeed, this has spurred the development of agents such as BMH-21, which can non-covalently bind GC-rich DNA, degrade Pol I and does not induce DNA damage.^42,286^ In this review, we focus on CX-5461, a novel anticancer agent that is showing increasing promise in clinical investigations.^287^

#### CX-5461: A first in class selective inhibitor of Pol I transcription

CX-5461 selectively inhibits Pol I transcription relative to Pol II and Pol III transcription^279,288^ and has demonstrated single-agent therapeutic efficacy in multiple preclinical cancer models, including lymphoma, AML, breast, prostate, and ovarian cancer.^39,43,289–295^

**Fig. 4:**
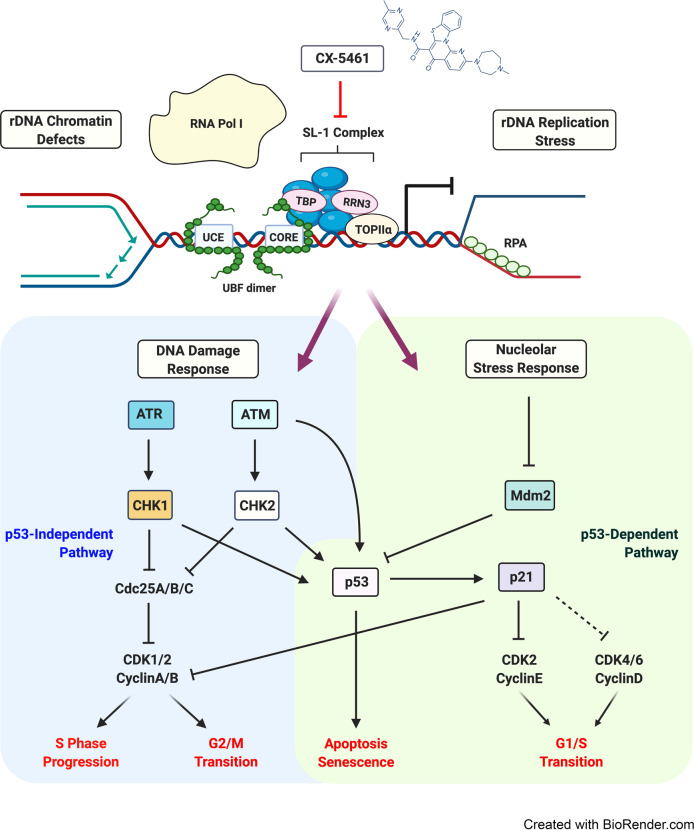
Inhibition of RNA polymerase I transcription by CX-5461 induces both p53-dependent and -independent responses. A schematic representation of CX-5461’s mode of action and its downstream stress response pathways. CX-5461 inhibits the initiation of Pol I-mediated transcription by disrupting the association between SL-1 and Pol I, thus preventing Pol I recruitment to the rDNA promoter. This displacement leads to “exposed” rDNA repeats devoid of Pol I and the presence of defects associated with an open chromatin structure and the recruitment and phosphorylation of RPA to single-stranded rDNA, a marker for replication stress. CX-5461-mediated alterations in rRNA synthesis and rDNA chromatin and topology in turn trigger the downstream activation of two major signaling pathways: (*i*) a canonical p53-dependent nucleolar stress response leading to accumulation of p53 and/or (*ii*) a p53-independent DNA damage response (DDR) involving the activation of ATM/ATR kinase signaling. Each pathway induces various cellular responses including G1/S and G2/M cell cycle defects, apoptosis and senescence. Pol I, RNA polymerase I; SL-1, selectivity factor 1; rDNA, ribosomal RNA gene; TBP, TATAbinding protein; UBF, upstream binding factor; UCE, upstream control element; RRN3, RNA polymerase I-specific transcription initiation factor; Mdm2, mouse double minute 2; CHK, checkpoint kinases; CDK, cyclin-dependent kinases; ATM indicates ataxia telangiectasia mutated; ATR, ataxia telangiectasia and Rad3-related

Using genetically engineered models of *Eμ-Myc* lymphoma, Bywater *et al*. demonstrated that CX-5461 induces nucleolar disruption, resulting in the binding of unassembled RPL5 and RPL11 to Mdm2 and subsequently rapidly activating p53-mediated cell cycle arrest and apoptosis^39^ (Fig. 4). Furthermore, this response was triggered in the absence of changes in the total levels of functional ribosomes or the rates of protein synthesis, demonstrating that certain tumor cells are highly dependent on accelerated Pol I transcription levels.^28^ The in vivo doses of CX-5461 that activate p53 and induce p53-mediated apoptotic cell death in MYC-driven B-cell lymphoma cells did not cause deleterious genotoxic effects to normal B-cell populations of the same lineage.^39^ Furthermore, a strong correlation between the mutational status of p53 and the level of drug sensitivity was also found, whereby p53-wildtype lymphoma and AML cell lines were significantly more sensitive towards CX-5461 than p53-null or -mutant cell lines.^39,291^ Inhibition of Pol I transcription with low-dose actinomycin D treatment and subsequent activation of the nucleolar stress response was also shown to delay the in vivo growth of p53-wildtype *Eμ-Myc* lymphomas but had no impact on the expansion of p53-null *Eμ-Myc* lymphomas.^296^ Nevertheless, CX-5461 demonstrated significant efficacy in p53-null AML in vivo. The significant survival advantage in both p53-wildtype and p53-null leukemic mice treated with CX-5461 was associated with activation of the G2/M cell cycle checkpoint, induction of myeloid differentiation and targeting of the leukemia-initiating cell population.^291^

P53-independent responses to CX-5461 in acute lymphoblastic leukemia (ALL), human ovarian cancer models and human immortalized fibroblasts were shown to involve a G2/M checkpoint arrest and cell death via the ATM/ ATR kinase pathways.^43,292,297,298^ In addition to inhibiting Pol I transcription, CX-5461 was shown to induce perturbations in rDNA chromatin and replication stress, triggering non-canonical activation of ATM/ATR kinase signaling within the nucleoli. Strikingly, these occurred in the absence of detectable γH2AX foci, a marker of double-stranded DNA breaks (DSBs)^298^ (Fig. 4). The net results of the unique CX-5461-mediated unique DNA damage response is replication stress, leading to replication-dependent DNA damage and cell cycle arrest and cell death.^43,294^ The combination of CX-5461 with dual inhibition of CHK1/2 has been reported to significantly enhance the therapeutic outcome against p53-null MYC-driven lymphoma in vivo.^36^

The first-in-human phase I dose escalation study with CX-5461 was recently completed in 16 patients with advanced relapsed or refractory hematological malignancies at the Peter MacCallum Cancer Centre, Australia (Australia and New Zealand Clinical Trials Registry, #12613001061729). CX-5461 was found to be both safe and tolerable at doses associated with a clinical benefit, with overall manageable dermatologic side effects.^287^ Clinical efficacy was also identified in ~30% (6/16) of the patients. In particular, one patient with anaplastic large-cell lymphoma had a prolonged partial response for >12 months, and 5 patients with either diffuse large B-cell lymphoma or multiple myeloma achieved periods of disease stabilization for varying lengths of time.

In addition to hematological cancers, a phase I/II clinical trial is also currently being conducted by the Canadian Cancer Trials Group (CCTG) in patients with advanced solid tumors characterized by DNA-repair deficiencies (NCT02719977, opened May 2016). CX-5461 thus far has been found to be well tolerated with 4 patients achieving a partial response and an additional 6 patients maintaining stable disease.^299^ Preliminary activity for CX-5461 has been observed in patients with homologous recombination (HR)-deficient tumors. An expansion cohort for patients with metastatic breast cancer with confirmed HR deficiency is currently open.

#### Sensitivity and resistance to ribosome-targeting therapy

While CX-5461 represents an exciting therapeutic option for multiple cancer types, the identification of predictive biomarkers of response to identify patients who will benefit from this therapy is essential for further clinical development. In a study utilizing a panel of ovarian cancer cell lines, sensitivity to CX-5461 was shown to be associated with a high baseline rate of Pol I transcription and higher proportion of active to inactive rDNA repeats, and independent of p53 mutation status.^43,44^ This is consistent with CX-5461’s mode of action in inhibiting Pol I transcription and triggering defects associated with open chromatin and replication stress at the rDNA leading to activation of p53-independent DDR.^295,298^ Therefore, cancer cells with a higher proportion of active rDNA are more sensitive to CX-5461-mediated nucleolar DDR and activation of cell cycle checkpoints.

Moreover, biomarkers of sensitivity to CX-5461 in ovarian cancer models include BRCA-mutated and MYC targets gene expression signatures that were found to be enriched in a subset of primary and relapsed ovarian cancer.^43^ As MYC is a master regulator of ribosome biogenesis, MYC-driven Pol I transcription and/or MYC-driven global transcription and replication stress may underlie sensitivity to CX-5461. In a recent genome-wide siRNA screen in ovarian cancer cells treated without or with CX-5461, a number of genes known to be important in HR DNA repair and other DNA repair pathways were identified to be synthetic lethal with CX-5461.^295^ The loss of DNA topoisomerase I (TOP1) was shown to cooperate with CX-5461 in inhibiting cell proliferation. TOP1 plays an important role in resolving topological stress at the rDNA loci,^300,301^ and TOP1 inhibitors such as topotecan have been used as a salvage therapy for relapsed ovarian cancer patients^302^ though their clinical use has been limited due to hematological toxicity. The combination of CX-5461 and low-dose topotecan markedly enhanced nucleolar replication stress and the DDR without enhancing DNA damage levels compared to single-agent treatment, leading to cell cycle arrest and decreased clonogenic survival of cancer cells.^295^ When CX-5461 and low-dose topotecan were combined to treat HGSOC tumors in vivo, significant inhibition of tumor growth occurred without observing side effects.^295^ This suggests that the toxicity often associated with using standard topotecan doses could potentially be lessened by using lower doses when combined with CX-5461.

CX-5461 has shown efficacy in combination with other therapies targeting ribosome biogenesis in a number of MYC-driven cancer models.^290,293^ PIM kinase is co-elevated in MYC-driven prostate cancer^303^ and is responsible for increasing MYC transcriptional activity and stability, and increasing translational activity via stimulation of phosphorylation of 4E-BP1. Elevation of PIM kinase has also been found to correlate with therapeutic resistance in prostate cancer. CX-5461 in combination with a PIM kinase inhibitor demonstrated marked therapeutic benefit compared to single-agent treatment, highlighting the promising therapeutic benefit in effective targeting of ribosome biogenesis in prostate cancer.^293^

It is well accepted that combinatorial targeting of multiple growth signaling pathways and/or processes required for cell growth and proliferation can prove effective in cancer treatment. Targeting of the PI3K/mTOR pathway upstream of ribosome biogenesis is not sufficient for robust clinical responses in many tumor types, due to feedback loops that maintain pathway activity and compensatory activation of RAS signaling,^45^ which also regulates ribosome biogenesis. Therefore, targeting the signaling networks that controls ribosome synthesis and function at multiple steps can improve therapeutic efficacy downstream of these oncogenic networks and delay the onset of acquired resistance.^45^ Indeed, CX-5461 in combination with everolimus, another inhibitor of mRNA translation that targets mTORC1 has shown remarkable therapeutic efficacy in the *Eμ-Myc* lymphoma murine model,^290^ with the combination therapy being well tolerated and demonstrating a prolonged survival benefit compared to single-agent therapy, with no negative effects on the wild-type B-cell population. Thus, multi-pronged targeting of ribosome synthesis and function at different steps or in combination of therapeutic targeting of upstream signaling pathways can provide substantial improvement in the efficacy of ribosome-targeting therapy. Together, the effective targeting of ribosome biogenesis and protein synthesis may prove effective in overcoming growth signaling pathway redundancies and tumor heterogeneity and can potentially be used for the treatment of a large subset of human cancer with RP gene deletions.^25^

In addition to activation of the nucleolar stress response and pro-apoptotic responses, inhibition of ribosome biogenesis by CX-5461 and everolimus combination therapy impairs mRNA translation capacity.^304^ Global translation profiling and metabolomic analysis have shown that the marked improvement in the in vivo efficacy of CX-5461 and everolimus combination therapies is associated with specific suppression of translation of mRNAs encoding regulators of cellular energetic metabolism.^304^ Consistent with the critical role of suppressed translation in therapeutic efficacy, acquired resistance to this co-treatment is driven by translational rewiring that results in deregulated mitochondrial respiration. Inhibition of mitochondrial function and energy biosynthesis with metformin, a widely used anti-diabetic agent, re-sensitized the resistant B-cell lymphoma cells to CX-5461 and everolimus treatment. These discoveries reveal that translational plasticity drives resistance to ribosome-directed therapy, expanding our understanding of ribosome addiction in cancer cells and reprogramming of mRNA translation and protein synthesis that drives drug resistance.

**Fig. 5:**
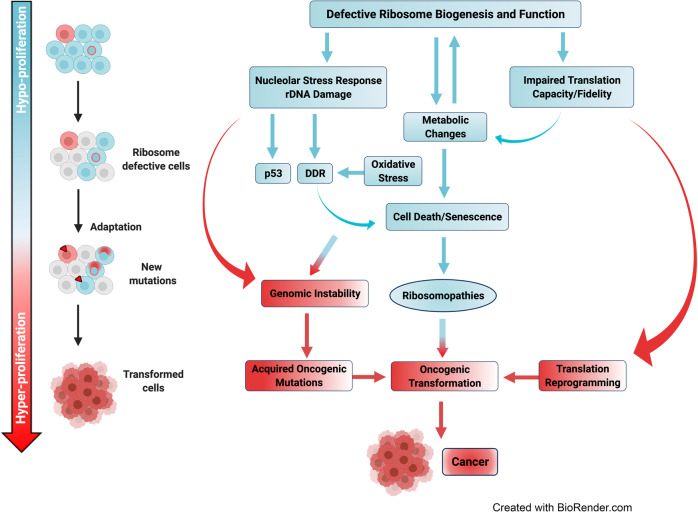
A model of the transition from cellular hypo-proliferation to hyper-proliferation in ribosomopathies. Defects in ribosome biogenesis and ribosome function induce p53 activation via the nucleolar stress response, but also activate the p53-independent DNA damage response (DDR). Defects in ribosome biogenesis also result in the selective translation of subsets of mRNAs involved in the regulation of cellular metabolism. In turn, deregulation of metabolism leads to oxidative stress that further impairs ribosome biogenesis and ribosome function. These nucleolar and metabolic stresses result in hypo-proliferative responses including cell cycle arrest, senescence or apoptosis that parallel the hypo-proliferative phenotypes associated with ribosomopathies. Chronic deregulation of ribosome biogenesis and cellular metabolism promotes genomic instability and secondary mutations, leading to the outgrowth of clones harboring translationally driven elevated metabolism and pro-survival mechanisms that underpin the transition from hypo-proliferation to hyper-proliferation phenotypes and cancer predisposition in ribosomopathies

These findings have direct relevance to understanding cancer predisposition in patients with ribosomopathies and provide an understanding of the adaptive molecular alterations that enable the switch from a hypo-proliferative to hyper-proliferative state. The data substantiate an evolutionary model of ribosomopathies whereby chronically compromised ribosome biogenesis results in the evolution of subclones with altered translation of a select subset of transcripts and/or acquired mutations that promote pro-survival mechanisms including translationally driven elevated metabolism and escape from cell cycle arrest/senescence (Fig. 5).

## Conclusions and perspectives

Genetic defects such as mutations in RPs as well as therapy-induced defects in ribosome biogenesis result in hypo-proliferative phenotypes, including cell cycle arrest, senescence, or apoptosis. The impairment of ribosome biogenesis, at any step from rRNA synthesis to ribosome assembly induces p53 activation via the nucleolar stress response and activates the DDR and other p53-independent stress pathways. Thus, combination therapeutic approaches that target signaling networks at multiple steps upstream of ribosome synthesis and function can provide potent and effective treatment options for oncogene-driven cancers.

Defects in ribosome biogenesis also result in an altered pattern of mRNA translation due to impaired translation capacity and fidelity, which may also contribute to the hypo-proliferative phenotypes associated with ribosomopathies. Oxidative stress and other metabolic changes exacerbate ribosome stress and induce DNA damage, promoting secondary mutations and genomic instability. The outgrowth of clones with enhanced survival capacity due to genetic mutations, translatomic and metabolic changes under the pressure of chronically compromised ribosome biogenesis, may underpin the transition from hypo-proliferative to hyper-proliferative phenotypes. Such selection may lead to malignant transformation in ribosomopathy patients and development of resistance to ribosome-targeting therapy. Thus, systematic and comprehensive analyses of the changes in mRNA translation, genetic mutations and metabolic alterations in response to RP mutations and compromised ribosome biogenesis will enable the identification of new vulnerabilities for RP mutation-associated diseases.
